# Slow walking on a treadmill desk does not negatively affect executive abilities: an examination of cognitive control, conflict adaptation, response inhibition, and post-error slowing

**DOI:** 10.3389/fpsyg.2015.00723

**Published:** 2015-05-27

**Authors:** Michael J. Larson, James D. LeCheminant, Kaylie Carbine, Kyle R. Hill, Edward Christenson, Travis Masterson, Rick LeCheminant

**Affiliations:** ^1^Department of Psychology and Neuroscience Center, Brigham Young University, ProvoUT, USA; ^2^Department of Exercise Sciences, Brigham Young University, ProvoUT, USA

**Keywords:** cognitive control, treadmill, work, conflict adaptation, conflict, exercise

## Abstract

An increasing trend in the workplace is for employees to walk on treadmills while working to attain known health benefits; however, the effect of walking on a treadmill during cognitive control and executive function tasks is not well known. We compared the cognitive control processes of conflict adaptation (i.e., congruency sequence effects—improved performance following high-conflict relative to low-conflict trials), post-error slowing (i.e., Rabbitt effect), and response inhibition during treadmill walking (1.5 mph) relative to sitting. Understanding the influence of treadmill desks on these cognitive processes may have implications for worker health and productivity. Sixty-nine individuals were randomized to either a sitting (*n* = 35) or treadmill-walking condition (*n* = 34). Groups did not differ in age or body mass index. All participants completed a computerized Eriksen flanker task and a response-inhibition go/no-go task in random order while either walking on a treadmill or seated. Response times (RTs) and accuracy were analyzed separately for each task using mixed model analysis of variance. Separate ANOVAs for RTs and accuracy showed the expected conflict adaptation effects, post-error slowing, and response inhibition effects when collapsed across sitting and treadmill groups (all *F*s > 78.77, *P*s < 0.001). There were no main effects or interactions as a function of group for any analyses (*F*s < 0.79, *P*s > 0.38), suggesting no decrements or enhancements in conflict-related control and adjustment processes or response inhibition for those walking on a treadmill versus sitting. We conclude that cognitive control performance remains relatively unaffected during slow treadmill walking relative to sitting.

## Introduction

Insufficient physical activity combined with prolonged sedentary time (often occurring in the workplace) may increase risk of chronic disease (e.g., Healy et al., 2011). Consequently, there is an increased need for workplace health interventions. Previous interventions include providing employees with information, workshops, and seminars on the benefits of healthy living (Mills et al., 2007), conducting team-based weight loss competitions (Morton et al., 2011), and emphasizing walking to work (Kitchen et al., 2011). Despite the benefits of multiple workplace programs, there remains a persistent need to improve work-related health behaviors (Tudor-Locke et al., 2014).

Activity programs centered on walking could be beneficial in this regard, since walking is a common physical activity that can help reduce cardiovascular disease, type-II diabetes, and obesity (Albright and Thompson, 2006; Sato et al., 2007). There are clear health benefits of walking at work (Kitchen et al., 2011); however, most participants in workplace health interventions are volunteers that are already motivated to change their behavior (Marshal, 2004). Therefore, programs and activities that increase the rate of participation in health interventions in the workplace need to be considered.

One such program in the workplace is for employees to utilize treadmill workstations (i.e., desks placed over treadmills that allow individuals to slowly walk while working). Walking on a treadmill at work has potential health benefits, such as increasing energy expenditure, alleviating stress, and reducing weight gain (Levine and Miller, 2007; Thompson et al., 2008); however, the effect of treadmill walking during work performance is not well known and limited research is currently available. Studies to date indicate that office tasks involving fine motor skills, such as typing and mouse performance, and solving math problems tend to decrease in proficiency when walking on a treadmill (John et al., 2009; Straker et al., 2009; Funk et al., 2012). If individuals are allowed to train on the treadmill desk, however, simple tasks such as typing performance are not as significantly affected (Thompson and Levine, 2011; Funk et al., 2012). Walking on a treadmill requires attentional cognitive resources that can increase response times (RTs) and decrease attention performance (Regnaux et al., 2006). The exception is when performing tasks that are relatively simple, then attention and processing speed seem to remain generally intact (John et al., 2009; Ohlinger et al., 2011; Alderman et al., 2014). The preponderance of research to date has utilized simple tests or focused primarily on tests of attention, but has rarely included measures that are sensitive to decrements in cognitive and executive control.

Cognitive control refers to the ability to guide one’s thoughts and actions in accord with internal goals (Miller and Cohen, 2001). Cognitive control is critical for identifying problematic patterns of behaviors, such as identifying mistakes and subsequently adjusting performance in order to accurately complete a task or improve workplace performance (Botvinick et al., 2001; Miller and Cohen, 2001). Cognitive control can be tested in the laboratory using tasks such as the Eriksen flanker task (Eriksen and Eriksen, 1974) or Stroop task (Stroop, 1935) that activate competing response options (e.g., asking participants to respond to the color of the word RED written in green ink on a Stroop task) or induce the commission of multiple errors. According to cognitive control theory, competing response options introduce conflict that is detected by the anterior cingulate cortex (ACC) that subsequently signals to the dorsolateral prefrontal (dlPFC) and/or ventrolateral prefrontal (vlPFC) cortices to introduce more attentional control and change behavior (Botvinick et al., 2001; Kerns et al., 2004; Egner, 2011; Larson et al., 2014).

Changes in cognitive control can be seen in slower RTs on trials following errors—frequently referred to as post-error slowing or the “Rabbitt” effect (Rabbitt, 1966)–or faster RTs on trials that are high in conflict (e.g., incongruent trials on a Stroop or flanker task) that follow a previous high-conflict trial, an effect also known as conflict adaptation, congruency sequence effects, or the “Gratton” effect (for ease of writing and interpretation we will refer to these as conflict adaptation effects hereafter: Gratton et al., 1992; Larson et al., 2009, 2014). More specifically, conflict adaptation shows the increased recruitment of cognitive control as evidenced by faster RTs on incongruent trials preceded by incongruent trials (iI trials) relative to incongruent trials preceded by congruent trials (cI trials; e.g., Gratton et al., 1992; Clayson and Larson, 2011). RTs and error rates also tend to increase on congruent trials preceded by an incongruent trial (iC trials) relative to congruent trials preceded by congruent trials (cC) trials due to switching between congruencies (Clayson and Larson, 2011).

In addition to looking at behavioral adjustments after errors and conflict, cognitive control is evident on trials when individuals must inhibit a response during a speeded task. For example, on a go/no-go task individuals are asked to respond as quickly and accurately when a stimulus (e.g., the letter “M”) is presented, but withhold their response when a less-frequently presented stimulus (e.g., the letter “W”) is presented. The accurate identification of a no-go trial and subsequent inhibition of response is consistently associated with activity of multiple areas of the prefrontal cortex (including the dlPFC and inferior frontal gyrus; Bari and Robbins, 2013).

Our knowledge of how cognitive control functions are affected while walking on a treadmill can be informed by the existing literature on the effects of acute exercise on cognitive performance generally and cognitive control. Broadly speaking, cognitive functioning has a small decline during the initial phases of performance with concurrent exercise (e.g., first 20 min), but has some improvements in rapid decision making and highly automatic tasks thereafter (Lambourne and Tomporowski, 2010). Other research clarifies that acute bouts of exercise are associated with small improvements in cognitive performance both during and after exercise, with the most benefit seen at moderate exercise intensity levels for cognitive performance speed (Chang et al., 2012; McMorris and Hale, 2012). Accuracy of cognitive performance is generally not affected by exercise at any intensity level (McMorris and Hale, 2012). Studies specific to the effects of exercise on cognitive control generally follow this same pattern. For example, Davranche et al. (2009) showed improved reaction times on cognitive control functions using a flanker task both during and following moderate intensity exercise. Similarly, studies in both children and older adults report some improvement in cognitive control performance following completion of a moderate exercise bout (Hillman et al., 2009; O’Leary et al., 2011; Drollette et al., 2012; Joyce et al., 2014). Research to date, however, has not focused on the effects of slow treadmill walking (low to very-low intensity), such as that in a workplace setting, on cognitive control-related adjustments in performance such as conflict adaptation and post-error slowing, as well as response inhibition processes.

The purpose of the current study, therefore, was to test the effects of slow treadmill walking (1.5 mph) on a treadmill desk, such as what might be used in the workplace, on cognitive control adjustment and response inhibition processes. Based on previous studies, we expected that groups would not differ on cognitive control processes, but may show some decrements on measures of response inhibition.

## Materials and Methods

### Participants

A total of 76 participants initially enrolled in the study. Upon arrival for the study, participants were randomly assigned using a random number generator to either a treadmill walking group (*n* = 37; 14 female) or a sitting group (*n* = 39; 22 female). A total of seven participants were excluded after study participation (four from the sitting group and three from the treadmill group). Participants were excluded due to computer malfunction wherein accuracy data were not recorded (*n* = 3) or due to poor task understanding leading to less than chance performance on either the go/no-go or flanker tasks (*n* = 4; two each from the treadmill and sitting groups—participants responded to no-go stimuli or to the flankers instead of the target). Thus, the final sample included 69 participants, with 34 (13 female) in the treadmill group and 35 (20 female) in the walking group. Ratio of females to males was slightly higher in the seated group, although this difference was not statistically significant, χ^2^(1) = 2.47, *p* = 0.12. All participants were healthy and did not differ in demographic or health-related variables, including age, years of education, body mass index (BMI), weight, or height (see **Table 1**). None of the participants had used treadmill desks previously and there were no practice or learning periods for the current tasks.

**Table 1:** Demographic and fitness characteristics of sitting and treadmill walking participants.

	Sitting *n* = 35	Treadmill *n* = 34	*t-*value	* p-*value
Age (yrs)	20.74 (2.12)	20.91 (2.43)	0.31	0.76
Weight (kg)	67.19 (12.13)	70.14 (13.52)	0.95	0.34
Height (cm)	172.26 (10.00)	173.89 (8.84)	0.72	0.47
Body mass index (BMI)	22.53 (2.81)	23.13 (4.03)	0.72	0.47

*Data include mean (SD).*

Participants provided written informed consent in accord with study procedures approved by the Brigham Young University Institutional Review Board. Participants were recruited from large undergraduate psychology courses and met the following inclusion criteria based on self-report: between the ages of 18 and 35, native English speakers, right handed, and self-reported normal or corrected-to-normal vision, and that they are able to jog for 7 min (to ensure sufficient physical fitness to complete the treadmill task). Participants were excluded if they were diagnosed with any chronic disease, psychiatric, or neurologic disorder (including any head injury with loss of consciousness or attention-deficit/hyperactivity disorder), were taking medication, were pregnant or currently lactating, had inconsistent sleep patterns, had a physical injury, or disability that prevented them from walking or jogging, or endorsed alcohol, nicotine, or substance abuse within the last year on a self-report measure. Participants either received course credit or financial compensation for their participation.

### Procedures

All participants completed the study individually between the hours of 7 and 10 am to control for possible variations due to time of day (Larson et al., 2015). The testing session lasted approximately 1 h. In an effort to control potential variation in energy due to caloric intake, all participants arrived in a fasted state having refrained from eating food or caloric beverage after 9 pm the previous night. Participants also indicated they had at least 7 h of sleep the previous night and had not used caffeine or participated any vigorous exercise activities for 24-h prior to testing. Upon arrival, inclusion/exclusion criteria were verbally confirmed, the informed consent was completed, and participants were assigned to the sitting or treadmill group. Subsequently, demographic information was gathered, body weight was measured using a digital scale (Tanita Corporation, Japan), and height was measured using a wall-mounted stadiometer (Seca, Chino, CA, USA).

Individuals assigned to the treadmill group received instruction on treadmill safety. They then completed a familiarization period of approximately 5 min of slow treadmill walking prior to beginning the study tasks. Responses for all tasks were given by pressing the correct keys on a typical QWERTY keyboard while viewing the tasks on a 17-inch computer monitor. Participants in the treadmill walking condition completed the tasks using a computer monitor and keyboard placed on a desk that was 37 inches wide and 24 inches deep placed over the top of the treadmill console. Participants in the sitting condition used the same monitor and keyboard while seated on a typical desk setup. The treadmill was a motor-driven treadmill (LifeSpan, Salt Lake City, UT, USA) programmed to a continuous speed of 1.5 miles per hour with a 0% grade. Tasks were presented in random order along with some additional memory, typing, and attention tasks (not included in the current manuscript, but presented in Larson et al., 2015) to control for potential order effects. Participants completed both a modified flanker task and a go/no-go task.

### Modified Flanker Task

Participants completed a modified version of the Eriksen flanker task (Eriksen and Eriksen, 1974) to examine conflict processing, conflict adaptation, and post-error slowing. Trials consisted of incongruent (e.g., <<><<) or congruent (e.g., <<<<<) arrow stimuli presented in 32-point Arial white font on a black background. Participants responded as quickly and accurately as possible to the direction of the middle (“target”) arrow with a right hand key press. Participants used an index finger button press for arrows pointing to the left and a middle finger button press for arrows pointing to the right. Flanking arrows were presented for 100 ms prior to the onset of the target stimulus that was on the screen for 600 ms (participants had up to 1500 ms to respond). Participants were shown a fixation cross during the inter-trial interval that varied randomly between 800, 1,000, and 1200 ms, with a mean of 1,000 ms. There were three blocks of 200 trials (600 total trials). Participants were allowed to rest for as long as they desired between blocks. The ratio of congruent to incongruent trials was 50/50.

### Go/No-Go Task

A go/no-go task was used to measure response inhibition. Participants were presented either the letter “M” or letter “W” in 36-point black Arial font on a white background. Letters were presented for 100 ms. A fixation cross was presented during an inter-trial interval that varied randomly between 300 and 800 ms to reduce expectancy effects of the next stimulus. Participants were instructed to respond with an index finger button press when presented with the letter “M” (a Go trial) and withhold their response when presented the letter “W” (a No-Go trial). The task consisted of five blocks of fifty trials with 40 Go trials and 10 No-Go trials (250 total trials, 200 Go trials, and 50 No-Go trials). From the task, we calculated the rate of No-Go response-inhibition errors (i.e., pressing a button during a No-Go trial).

### Data Analysis

Data were analyzed separately for the flanker and go/no-go tasks. For conflict adaptation analyses, RT data were calculated excluding error and post-error trials, as these are associated with impulsive (i.e., fast) or slowed RTs, respectively. Mean RTs and accuracy were analyzed using separate 2-Group (treadmill, sitting) × 2-Previous-trial Congruency (congruent, incongruent) × 2-Current-trial Congruency (congruent, incongruent) repeated measures analyses of variance (ANOVA). Post-error slowing was examined using only correct trials and comparing post-correct trial RTs and post-error trials RTs using a 2-Group × 2-Accuracy (post-correct, post-error) ANOVA. We used tests of simple effects to decompose significant main effects and interactions. Partial-eta^2^ ($\eta_{p}^{2}$) is reported as a measure of effect size for ANOVA analyses. For the go/no-go data, we focused on no-go response-inhibition errors comparing groups using independent samples *t*-tests. Cohen’s *d* is presented as a measure of effect size. We examined homogeneity of variance for all variables. When sample variance was heterogenous, the Levene’s test of equality of variances is noted and a *t*-test not assuming equal variances is reported.

## Results

### Conflict Adaptation

#### Reaction Times

Response time and accuracy data for the sitting and treadmill groups for conflict adaptation and post-error slowing is presented in **Table 2**. The Group × Previous-trial Congruency × Current-trial Congruency ANOVA showed a significant main effect of current-trial congruency, *F*(1,67) = 839.77, *p* < 0.001, $\eta_{p}^{2}$ = 0.93, with the expected slower RTs to incongruent versus congruent trials. The Previous-trial Congruency × Current-trial Congruency interaction was also significant, *F*(1,67) = 147.03, *p* < 0.001, $\eta_{p}^{2}$ = 0.69, indicating there was significant conflict adaptation when collapsed across groups. As expected, cI trials were significantly slower than iI trials, *t*(68) = 5.32, *p* < 0.001, and iC trials were slower than cC trials, *t*(68) = 13.69, *p* < 0.001. Most important, however, the Group × Current-trial Congruency, *F*(1,67) = 3.19, *p* = 0.08, $\eta_{p}^{2}$ = 0.05, Group × Previous-trial Congruency × Current-trial Congruency, *F*(1,67) = 0.08, *p* = 0.78, $\eta_{p}^{2}$ = 0.001, and the main effect of group, *F*(1,67) = 0.77, *p* = 0.38, $\eta_{p}^{2}$ = 0.01, were not significant—indicating no significant group-related differences on conflict adaptation-related RTs.

**Table 2:** Cognitive control and response inhibition outcome variables for sitting and treadmill walking participants.

	Sitting (*n* = 35)	Treadmill (*n* = 34)
Congruent RT	405.38 (43.71)	418.24 (39.70)
Incongruent RT	479.24 (41.03)	483.72 (43.77)
cC RT	394.06 (41.76)	408.40 (40.64)
iC RT	417.00 (46.49)	428.50 (39.52)
cI RT	483.72 (45.42)	488.79 (48.07)
iI RT	474.92 (37.96)	478.57 (39.86)
Congruent accuracy	98% (2%)	98% (2%)
Incongruent accuracy	92% (5%)	93% (5%)
cC accuracy	98% (2%)	98% (2%)
iC accuracy	98% (3%)	97% (2%)
cI accuracy	90% (6%)	91% (6%)
iI accuracy	94% (4%)	95% (4%)
Post-correct RT	441.38 (41.61)	450.35 (40.10)
Post-error RT	488.71 (67.69)	501.64 (69.70)
Go accuracy	84% (12%)	84% (11%)
No-Go accuracy	67% (14%)	67% (14%)

*Data include mean (SD).*

*All response time (RT) data are in milliseconds. cC, congruent trial preceded by congruent trial; iC, congruent trial preceded by incongruent trial; cI, incongruent trial preceded by congruent trial; iI, incongruent trial preceded by incongruent trial.*

#### Accuracy

Accuracy data were very similar to the RT data. As expected, there was a significant main effect of Current-trial congruency, *F*(1,67) = 106.70, *p* < 0.001, $\eta_{p}^{2}$ = 0.61, and significant Previous-trial Congruency × Current-trial Congruency interaction, *F*(1,67) = 78.77, *p* < 0.001, $\eta_{p}^{2}$ = 0.54. Performance was worse on cI trials relative to iI trials, *t*(68) = 9.40, *p* < 0.001, and iC trials relative to cC trials, *t*(68) = 2.02, *p* = 0.05, indicating that the expected congruency and conflict adaptation effects were present. There was not a significant Group × Current-trial Congruency, *F*(1,67) = 0.54, *p* = 0.47, $\eta_{p}^{2}$ = 0.008, Group × Previous-trial Congruency × Current-trial Congruency, *F*(1,67) = 0.01, *p* = 0.94, $\eta_{p}^{2}$ < 0.001, or main effect of group, *F*(1,67) = 0.04, *p* = 0.84, $\eta_{p}^{2}$ < 0.001.

### Post-Error Slowing

The Group × Accuracy ANOVA revealed that post-error slowing was present when collapsed across groups, with a significant main effect of accuracy, *F*(1,67) = 78.77, *p* < 0.001, $\eta_{p}^{2}$ = 0.54. The Group × Accuracy interaction, *F*(1,67) = 0.12, *p* = 0.73, $\eta_{p}^{2}$ = 0.002, and the main effect of group, *F*(1,67) = 0.79, *p* = 0.38, $\eta_{p}^{2}$ = 0.01, were not significant—indicating similar post-error RTs between groups.

### Go/No-Go

Response inhibition accuracy for both go and no-go trials is presented in **Table 2**. Sitting and walking groups performed nearly identically on both go-trial, *t*(67) = 0.25, *p* = 0.81, *d* = 0.03, and no-go response-inhibition accuracy, *t*(67) = 0.03, *p* = 0.98, *d* < 0.001.

## Discussion

We aimed to examine the effects of concurrent slow treadmill walking on the cognitive and executive control functions of conflict adaptation, post-error slowing, and response inhibition. Consistent with expectations, conflict adaptation and post-error slowing were evident when collapsed across groups. Notably, however, there were no significant differences between individuals in the walking and sitting conditions for any cognitive control or response inhibition measures. Findings are consistent with another recent study of treadmill desks that focused on cognitive and executive control functions (Alderman et al., 2014). These authors indicated that treadmill-type workstations are one plausible way to address health issues related to sedentary lifestyle rates based on their findings and previous studies showing treadmill desks offer increased energy expenditure and health benefits relative to both sitting and other forms of work-related movement such as sit-stand desks (Levine and Miller, 2007; Thompson et al., 2008; Tudor-Locke et al., 2014). Our findings, using tasks associated with the cognitive control functions of post-error slowing, conflict adaptation, and inhibition of inappropriate response tendencies, support Alderman’s conclusion.

Other studies focusing on additional workplace abilities, such as fine motor skills, attention, and working memory are somewhat more mixed about the effects of treadmill desks in the workplace. For example, our group recently found that relative to sitting controls, treadmill walking decreased typing performance, was associated with decreased learning (but intact longer-term memory), and reduced attention/working memory (Larson et al., 2015). Other treadmill workstation studies also report poorer typing performance when walking relative to when seated (John et al., 2009; Straker et al., 2009; Thompson and Levine, 2011; Funk et al., 2012). In contrast, three other studies are consistent with the current findings showing treadmill walking generally does not affect measures of attention, processing speed, and cognitive control (John et al., 2009; Ohlinger et al., 2011; Alderman et al., 2014). The common component amongst the studies that show no significant differences between groups is the presence of executive/cognitive control measures. Specifically, the current study used the flanker test and a go/no-go test. John et al. (2009), Ohlinger et al. (2011), and Alderman et al. (2014) all used the Stroop color–word test (another index of attention and cognitive control) as well as the flanker test similar to that used in this study (Alderman et al., 2014), and the Auditory Consonant Trigrams test (a measure of attention and working memory; Ohlinger et al., 2011). Taken together, results suggest that executive and cognitive control measures remain similar to sitting performance when walking on a treadmill, whereas typing (fine motor skills) and potentially speed of learning and data manipulation are reduced. Studies replicating these results and examining multiple cognitive domains, including learning, memory, language, visuospatial functioning, etc. are needed to further understand the impact of workplace treadmill walking on cognitive and work performance.

Our findings of no specific decreases in cognitive control and response inhibition functioning during treadmill walking can be contrasted with previous research showing improvements in cognitive control associated with fitness and exercise. For example, Davranche et al. (2009) showed improved Flanker performance during cycling. Meta-analytic work looking at the speed and accuracy of cognitive functioning suggests that increased arousal during moderate-intensity exercise is associated with faster processing speed, but not improved accuracy (McMorris and Hale, 2012). The low intensity exercise used in workplace situations likely does not induce sufficient arousal to improve reaction time performance in accord with an inverted-U interpretation. More important, however, is the finding that a workplace-type treadmill desk does not impair cognitive control conflict or error identification and subsequent adjustment processes as well as response inhibition, which can be impaired at more moderate intensity exercise levels (Davranche and McMorris, 2009).

Our study should be understood within the context of several limitations and strengths. We include one of the largest sample sizes amongst treadmill workstation studies to date; however, the use of a two-group randomized design may have introduced unnecessary between-groups variability into the results. The absence of between-groups findings should be further examined in future studies using within-subjects, counterbalanced designs. We also used healthy university students that may not completely generalize to other workplace populations. Next, there was no baseline testing or practice trials provided. Whereas it is unlikely that groups of healthy college students differed in baseline cognitive functioning, it is possible and should be considered. The absence of practice trials may have increased learning effects, but these effects would have been the same between groups. In addition, we only used one speed of walking. The beneficial effects of exercise on task processing speed (but not accuracy) appear to be maximized at moderate levels of exercise intensity (McMorris and Hale, 2012) and the optimal speed for walking during workplace activities is unknown, although one study suggests 2.25 km/hr (1.4 mph) results in optimal typing performance (Funk et al., 2012). Using the same speed of walking may have also been differentially taxing for different individuals (e.g., individuals with different fitness levels or length of legs); however, we chose to be consistent in the speed to ensure possible between-groups differences were not due to individual variation in speed of the treadmill. Future studies should incorporate multiple levels of intensity and collect heart rate data to test the effects of effort level on these findings. Notably, however, we were focused on low to very-low intensity walking rather than moderate or vigorous exercise. Finally, all study procedures were conducted between 7 and 10 am. It is possible that restricting the time of day may have artificially altered some participants’ typical lifestyle and day-to-day patterns.

In sum, our findings and those of previous studies on executive and cognitive control tasks using treadmill workstations suggest no detrimental effect of slow treadmill walking on performance. Other workplace tasks such as fine motor skills and learning may be detrimentally affected, but future research is needed to replicate these findings. We conclude, however, that cognitive control performance remains relatively unaffected during conditions of slow treadmill walking and that treadmill walking may provide a good way to increase energy expenditure with periodic use in the workplace environment.

## Conflict of Interest Statement

The authors declare that the research was conducted in the absence of any commercial or financial relationships that could be construed as a potential conflict of interest.
